# Predictive value of serum sortilin, HMGB1, and galanin-like peptide for gestational diabetes mellitus in women with polycystic ovary syndrome

**DOI:** 10.3389/fendo.2025.1602622

**Published:** 2025-05-19

**Authors:** Hui Li, Xinghao Zhao

**Affiliations:** Department of Gynecology, Henan Provincial People’s Hospital, Zhengzhou University People’s Hospital, Zhengzhou, Henan, China

**Keywords:** polycystic ovary syndrome, gestational diabetes mellitus, biomarkers, HMGB1, galanin-like peptide

## Abstract

**Background:**

Polycystic ovary syndrome (PCOS) is associated with increased risk of gestational diabetes mellitus (GDM), but reliable early predictive biomarkers remain lacking. This study investigated the predictive value of serum Sortilin, HMGB1, and galanin-like peptide (GALP) for GDM development in PCOS pregnancies.

**Methods:**

This prospective cohort study enrolled 139 PCOS patients. Serum Sortilin, HMGB1 and GALP levels were measured by ELISA at 8-12 weeks. GDM was diagnosed at 24-28 weeks using 75g OGTT (IADPSG criteria). Predictive performance was assessed using multivariable logistic regression and receiver operating characteristic (ROC) curve analysis, with adjustment for maternal age, BMI, and lipid profiles.

**Results:**

The PCOS-GDM group (n=60) showed significantly higher levels of all biomarkers versus controls (n=79) (all p<0.001). GALP (aOR=1.55, 95%CI:1.05-1.92) and HMGB1 (aOR=1.65, 95%CI:1.50-1.79) independently predicted GDM after adjustment. The combined model achieved superior prediction (AUC=0.84, 95%CI:0.74-0.94) versus individual markers.

**Conclusion:**

Serum GALP and HMGB1 are promising early predictors of GDM in PCOS pregnancies, with combined assessment offering optimal risk stratification. These findings may facilitate timely intervention in high-risk populations.

## Introduction

Polycystic ovary syndrome (PCOS) is one of the most common endocrine and metabolic disorders affecting women of reproductive age, with a prevalence ranging from 5% to 15% (1–4). Characterized by heterogeneous clinical manifestations, PCOS typically presents with menstrual irregularities, chronic anovulation, hyperandrogenism, and insulin resistance (5–7). Beyond its reproductive implications, PCOS is associated with a spectrum of metabolic comorbidities, including type 2 diabetes mellitus, dyslipidemia, and increased cardiovascular risk, underscoring its long-term health burden (8–10). In the context of pregnancy, women with PCOS are at increased risk for a range of adverse pregnancy outcomes, such as early miscarriage, gestational hypertension, preeclampsia, and preterm delivery (11–13).

Gestational diabetes mellitus (GDM) is defined as the first onset or diagnosis of glucose metabolism abnormalities during pregnancy (14, 15). With the updating of diagnostic criteria, increasing proportion of advanced maternal age, and improved nutritional status, its incidence continues to rise. GDM poses significant threats to both maternal and fetal health in both short- and long-term perspectives (16–18). However, due to its insidious early symptoms, diagnosis typically relies on oral glucose tolerance tests during the second trimester, by which time adverse effects have already been established (19, 20). Therefore, early prediction and intervention are crucial. The pathogenesis of pregnancy complications in PCOS patients is currently believed to be closely associated with the interplay between endocrine dysfunction, chronic inflammatory state, and metabolic abnormalities (21–23). Studies demonstrate that PCOS, as a significant risk factor for GDM, is characterized by pre-existing endocrine and metabolic disturbances prior to pregnancy (13, 24). When superimposed with physiological hormonal changes and exacerbated insulin resistance during gestation, these women face a higher susceptibility to GDM (25, 26).

Despite growing understanding of these risks, reliable serological biomarkers for early pregnancy screening of high-risk populations remain lacking in clinical practice (22, 27). Early identification of GDM risk in PCOS patients is critical for implementing timely clinical interventions and improving perinatal outcomes (28, 29). Consequently, exploring and validating serum biomarkers capable of predicting GDM in this population has emerged as an urgent and significant clinical need. Early identification of GDM risk in PCOS pregnancies is clinically important, as it allows for timely interventions to prevent adverse maternal and fetal outcomes. This study focuses on investigating potential predictive biomarkers for GDM in PCOS pregnancies, aiming to establish an early warning system that could facilitate personalized clinical management strategies.

Recent studies have revealed that Sortilin, a member of the Vps10p receptor family, participates in glucose homeostasis regulation by modulating lipid metabolism and insulin signaling pathways (30, 31). Meanwhile, high-mobility group box 1 (HMGB1), as a critical proinflammatory mediator, exacerbates insulin resistance through activation of the TLR4/NF-κB pathway (32). Additionally, the neuroendocrine peptide galanin-like peptide (GALP) is involved in glucose metabolism regulation by influencing feeding behavior, energy metabolism, and insulin sensitivity (33, 34). Notably, PCOS patients exhibit characteristic endocrine-metabolic disturbances, which may lead to distinctive alterations in the expression profiles of these biomarkers in serum (17, 35).

However, the predictive value of combined detection of Sortilin, HMGB1, and GALP for assessing GDM risk in PCOS patients remains unclear, and the synergistic mechanisms underlying their roles in the progression from PCOS to GDM have not been systematically investigated. A comprehensive exploration of the dynamic changes in these biomarkers and their predictive potential for GDM development would not only contribute to elucidating the pathogenesis of PCOS-associated GDM but, more importantly, could provide novel molecular targets and predictive strategies for the early identification of high-risk individuals in clinical practice. Therefore, this study aims to evaluate the predictive value of serum Sortilin, HMGB1, and GALP levels measured in early pregnancy for the subsequent development of GDM in women with PCOS. The primary outcome is to determine whether these biomarkers independently or jointly predict GDM onset in this high-risk population. The secondary outcome is to explore the potential pathophysiological relevance and dynamic changes of these biomarkers during pregnancy in PCOS patients, thereby providing insights into novel mechanisms and early intervention strategies.

## Methods

### Study population

This prospective cohort study enrolled 139 pregnant women with PCOS who attended Henan Provincial People’s Hospital between May 2019 and December 2023. The study protocol was approved by the Institutional Review Board of Henan Provincial People’s Hospital, and all participants provided written informed consent. The sample size was determined based on previously reported GDM incidence rates in PCOS populations. Using a two-sample proportion comparison formula with α=0.05 and β=0.2 (power=80%), the minimum required sample size was calculated to be 120 subjects. Ultimately, 139 participants were recruited to ensure adequate statistical power, accounting for potential attrition.

### Inclusion and exclusion criteria

Inclusion criteria: (1) Age 18-40 years; (2) Diagnosis of PCOS according to the *Guidelines for the Diagnosis and Treatment of Polycystic Ovary Syndrome* (36); (3) Singleton pregnancy; (4) No pre-existing hypertension, diabetes mellitus, cardiovascular diseases, or other chronic medical conditions prior to pregnancy; (5) Regular antenatal care at our institution with complete follow-up data through at least 28 weeks of gestation.

Exclusion criteria: (1) Pre-pregnancy diagnosis of diabetes mellitus, hypertension, or major organ dysfunction; (2) Comorbid malignancies, neurological disorders, or severe psychiatric conditions; (3) Multiple gestation pregnancy; (4) Incomplete clinical data.

### Diagnostic criteria and methodology for GDM

This study employed standardized diagnostic criteria for GDM in accordance with the Guidelines for the Diagnosis and Treatment of Diabetes in Pregnancy (37). All participants underwent a 75g oral glucose tolerance test (OGTT) between 24-28 weeks of gestation following an 8-10 hour overnight fast, with venous blood samples collected at fasting, 1-hour, and 2-hour post-glucose load intervals. Plasma glucose levels were analyzed using an automated biochemical analyzer, with GDM diagnosis confirmed if any single threshold was met: fasting ≥5.1 mmol/L, 1-hour ≥10.0 mmol/L, or 2-hour ≥8.5 mmol/L. To ensure methodological rigor and data reliability, all procedures were conducted by uniformly trained laboratory personnel under strict quality control protocols, thereby minimizing variability and enhancing the clinical validity of the diagnostic outcomes. This standardized approach not only aligns with international consensus but also provides a robust foundation for investigating potential biomarkers and their association with GDM development in high-risk populations.

### Clinical data collection

Baseline clinical and biochemical information was collected at the time of enrollment. Demographic data included maternal age and pre-pregnancy body mass index (BMI), calculated as weight in kilograms divided by the square of height in meters (kg/m²). Fasting blood samples were also used to measure insulin levels and serum total testosterone using standardized laboratory methods at the institutional clinical laboratory. Gestational age was determined based on the last menstrual period and confirmed by first-trimester ultrasound.

### Measurement of serum biomarkers

Fasting venous blood samples (5 mL) were obtained from each participant during the first trimester of pregnancy, specifically between 8 and 12 weeks of gestation. Blood samples were collected using EDTA-containing tubes and immediately placed on ice. Within 30 minutes of collection, samples were centrifuged at 3000 rpm for 15 minutes at 4°C to separate the serum. The serum aliquots were stored at −80°C until batch analysis. Serum concentrations of Sortilin, high mobility group box 1 (HMGB1), and Galanin-like Peptide (GALP) were quantified using ELISA kits: Sortilin (Cat. No. EK1234, R&D Systems), HMGB1 (Cat. No. ST51011, Sigma-Aldrich), and GALP (Cat. No. CSB-EQ027578HU, Cusabio), following the manufacturer’s protocols. The minimum detection limits for Sortilin, HMGB1, and GALP, respectively. All samples were analyzed in duplicate to ensure reliability, and the intra-assay and inter-assay coefficients of variation were maintained below 10%. In a subset of samples, quantitative validation was performed using liquid chromatography–tandem mass spectrometry (LC-MS/MS) to confirm peptide specificity and concentration accuracy.

### Statistical analysis

All statistical analyses were performed using R software or SPSS Continuous variables were tested for normality using the Shapiro-Wilk test. Normally distributed data were expressed as mean ± standard deviation and compared using the Student’s t-test. Non-normally distributed data were presented as median and interquartile range (IQR), and compared using the Mann–Whitney U test. Correlations between serum biomarker levels and clinical parameters were assessed using Pearson or Spearman correlation coefficients, depending on data distribution. Univariate and multivariate logistic regression analyses were conducted to identify independent predictors of gestational diabetes mellitus (GDM). Multicollinearity among predictors was assessed using the variance inflation factor (VIF). The robustness of the multivariate models was tested by performing sensitivity analyses excluding outliers based on Cook’s distance. Internal validation of the model was performed using 10-fold cross-validation. Model calibration was examined using the Hosmer–Lemeshow goodness-of-fit test. The predictive performance of each biomarker, as well as their combined model, was evaluated using receiver operating characteristic (ROC) curve analysis. The area under the curve (AUC) and 95% confidence intervals (CIs) were calculated. Comparisons between AUCs were performed using DeLong’s test. A two-sided p-value < 0.05 was considered statistically significant.

## Results

### Comparative analysis of serum biomarkers in PCOS patients with and without GDM

**Figure 1:**
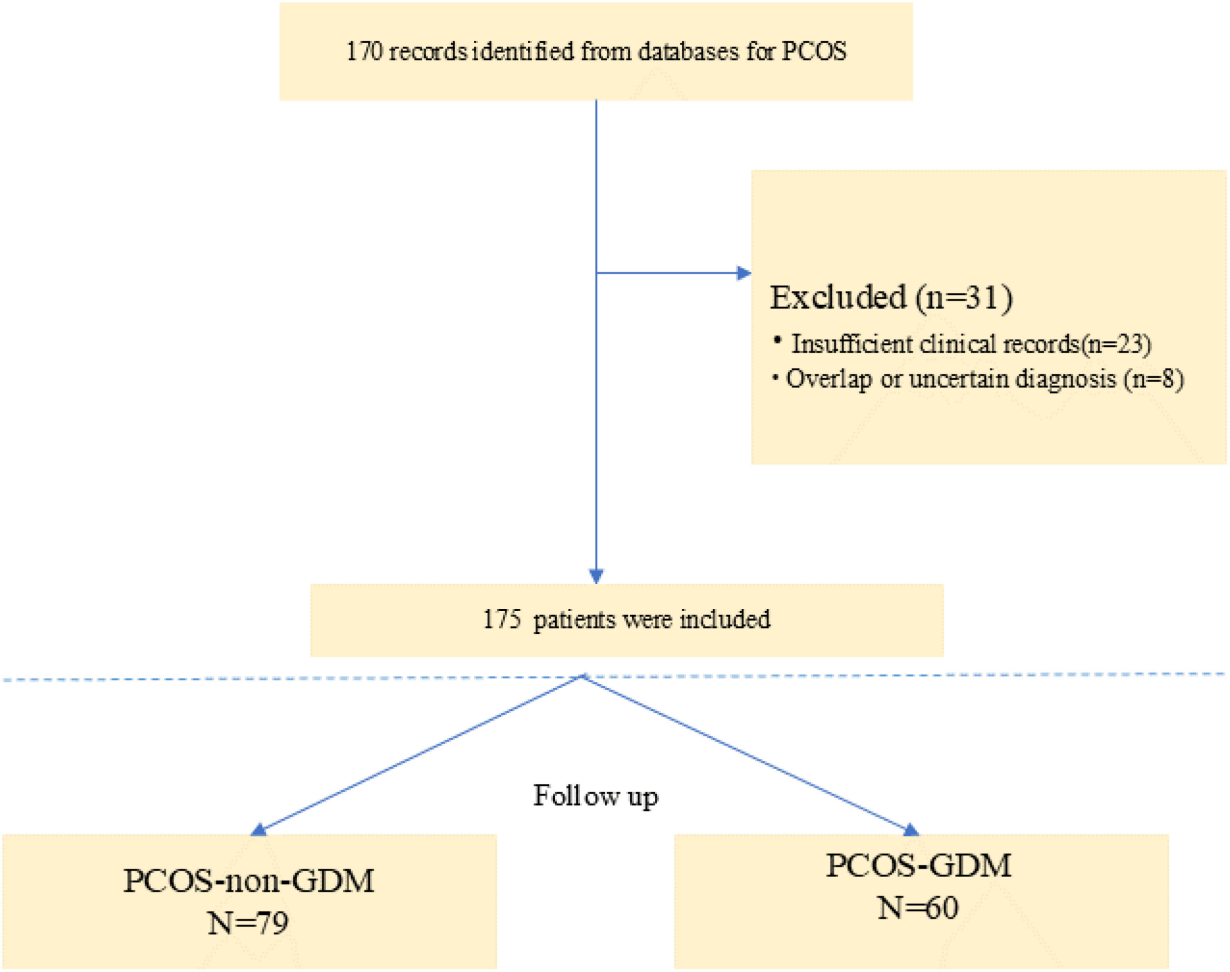
Study flow chart.

The study included a total of 139 baseline patients with PCOS who were prospectively followed during pregnancy (**Figure 1**). Based on oral glucose tolerance testing at 24-28 gestational weeks, participants were stratified into two groups: 79 women with PCOS-non-GDM (56.8%) and 60 women with PCOS-GDM (43.2%). As presented in **Table 1**, the demographic and biochemical characteristics were compared between groups. No significant differences were observed in age (32.36 ± 11.22 vs 33.17 ± 10.83 years, p=0.618), BMI (24.12 ± 3.29 vs 24.88 ± 2.91 kg/m², p=0.382), or obstetric history including gravidity (median=1.00 [IQR:1.00-2.00] for both groups, p=0.873) and parity (median=1.00 [IQR:1.00-2.00] for both groups, p=0.132). Conception methods showed similar distribution between groups (natural conception: 65.8% vs 68.3%; ART: 34.2% vs 31.7%, p=0.519). Notably, the PCOS-GDM group demonstrated significantly higher levels of all three investigated biomarkers compared to PCOS-non-GDM controls: GALP (88.92 ± 12.17 vs 41.63 ± 15.24 pg/mL, p<0.001), HMGB1 (76.81 ± 8.97 vs 52.71 ± 6.73 ng/mL, p<0.001), and Sortilin (242.31 ± 15.24 vs 172.42 ± 18.63 ng/mL, p<0.001). LH levels were also significantly elevated in the GDM group (13.27 ± 2.21 vs 11.31 ± 1.46 IU/L, p=0.006), while other hormonal and lipid parameters showed no statistically significant differences between groups.

**Table 1:** Demographic data and results of biochemical analyses.

Characteristics	PCOS-non-GDM (n=79)	PCOS-GDM (n=60)	*P* value
Age (years)	32.36 ± 11.22	33.17 ± 10.83	0.618
BMI (kg/m²)	24.12 ± 3.29	24.88 ± 2.91	0.382
Gravidity [times, Median (P25, P75)]	1.00(1.00, 2.00)	1.00(1.00, 2.00)	0.873
Parity [times, Median (P25, P75)]	1.00(1.00, 2.00)	1.00(1.00, 2.00)	0.132
Conception method (n, %)			0.519
Natural conception	52(65.8%)	41(68.3%)	
Assisted reproductive technology (ART)	27(34.2%)	19(31.7%)	
Gestational weeks at first prenatal visit	10.23 ± 7.14	10.82 ± 6.92	0.657
Gestational weeks at OGTT testing	25.21 ± 5.12	25.03 ± 4.83	0.219
Total cholesterol (mmol/L)	4.14 ± 0.82	4.26 ± 1.01	0.278
High-density lipoprotein cholesterol (HDL-C)	1.18 ± 0.13	1.24 ± 0.18	0.319
Low-density lipoprotein cholesterol (LDL-C)	2.96 ± 0.19	3.18 ± 0.67	0.217
Estradiol (ng/L)	95.89 ± 11.29	96.17 ± 11.92	0.789
Follicle-stimulating hormone (FSH, IU/L)	10.84 ± 2.56	9.56 ± 2.13	0.061
Luteinizing hormone (LH, IU/L)	11.31 ± 1.46	13.27 ± 2.21	0.006
GALP (pg/mL)	41.63 ± 15.24	88.92 ± 12.17	<0.001
HMGB1(ng/mL)	52.71 ± 6.73	76.81 ± 8.97	<0.001
Sortilin(ng/mL)	172.42 ± 18.63	242.31 ± 15.24	<0.001

GALP, Galanin-Like Peptide; HMGB1, High Mobility Group Box 1.

Data presentation: Mean ± SD or median (IQR), as appropriate.

Statistical tests: Student’s t-test, or chi-square test.

Significance level: Two-tailed p < 0.05.

### Risk factors for GDM development in PCOS patients

Logistic regression analysis revealed that elevated serum levels of GALP and HMGB1 were significantly associated with increased risk of GDM in women with PCOS (**Table 2**). In univariate analysis, GALP showed an OR of 1.693 (95%CI: 1.542-1.845, p=0.012), while HMGB1 demonstrated an even stronger association (OR 1.963, 95% CI 1.752-2.134, p=0.008). After adjusting for potential confounders including age, BMI and lipid profiles in multivariate analysis, both biomarkers remained statistically significant independent predictors: GALP maintained an adjusted OR (aOR) of 1.549 (95% CI 1.052-1.924, p=0.006) and HMGB1 showed an aOR of 1.647 (95% CI 1.502-1.793, p=0.015). Notably, Sortilin, while significant in univariate analysis (OR 1.721, 95% CI 1.598-1.845, p=0.009), lost statistical significance after adjustment (aOR 1.503, 95% CI 0.486-1.624, p=0.164). Traditional metabolic parameters including BMI (p=0.436), total cholesterol (p=0.241) and LDL-C (p=0.372) failed to show significant associations in either analysis.

**Table 2:** Risk factors for GDM development in PCOS patients.

Characteristics	Univariate Logistic Regression		Multivariate Logistic Regression	
	OR (95% CI)	P	OR (95% CI)	P
Age	1.842 (0.732-1.975)	0.187		
BMI	1.305 (0.412-1.487)	0.436		
Total cholesterol	1.672 (0.583-1.792)	0.241		
HDL-C	1.462 (0.473-1.597)	0.284		
LDL-C	1.427 (0.386-1.538)	0.372		
Estradiol	1.512 (0.497-1.629)	0.158		
FSH	1.884 (0.812-2.017)	0.213		
LH	1.236 (0.324-1.418)	0.527		
GALP	1.693 (1.542-1.845)	0.012	1.549 (1.052-1.924)	0.006
HMGB1	1.963 (1.752-2.134)	0.008	1.647 (1.502-1.793)	0.015
Sortilin	1.721 (1.598-1.845)	0.009	1.503 (0.486-1.624)	0.164

• GALP, Galanin-Like Peptide; HMGB1, High Mobility Group Box 1; HDL-C, High-Density Lipoprotein Cholesterol; LDL-C, Low-Density Lipoprotein Cholesterol; FSH, Follicle-Stimulating Hormone; LH, Luteinizing Hormone.

• The dependent variable in the logistic regression model is the development of GDM, diagnosed at 24–28 gestational weeks. The independent variables include baseline maternal characteristics (age, BMI), lipid profiles (total cholesterol, HDL-C, LDL-C), reproductive hormones (estradiol, FSH, LH), and serum biomarkers measured at 8–12 weeks of gestation (Sortilin, HMGB1, GALP).

### ROC curve analysis

**Figure 2:**
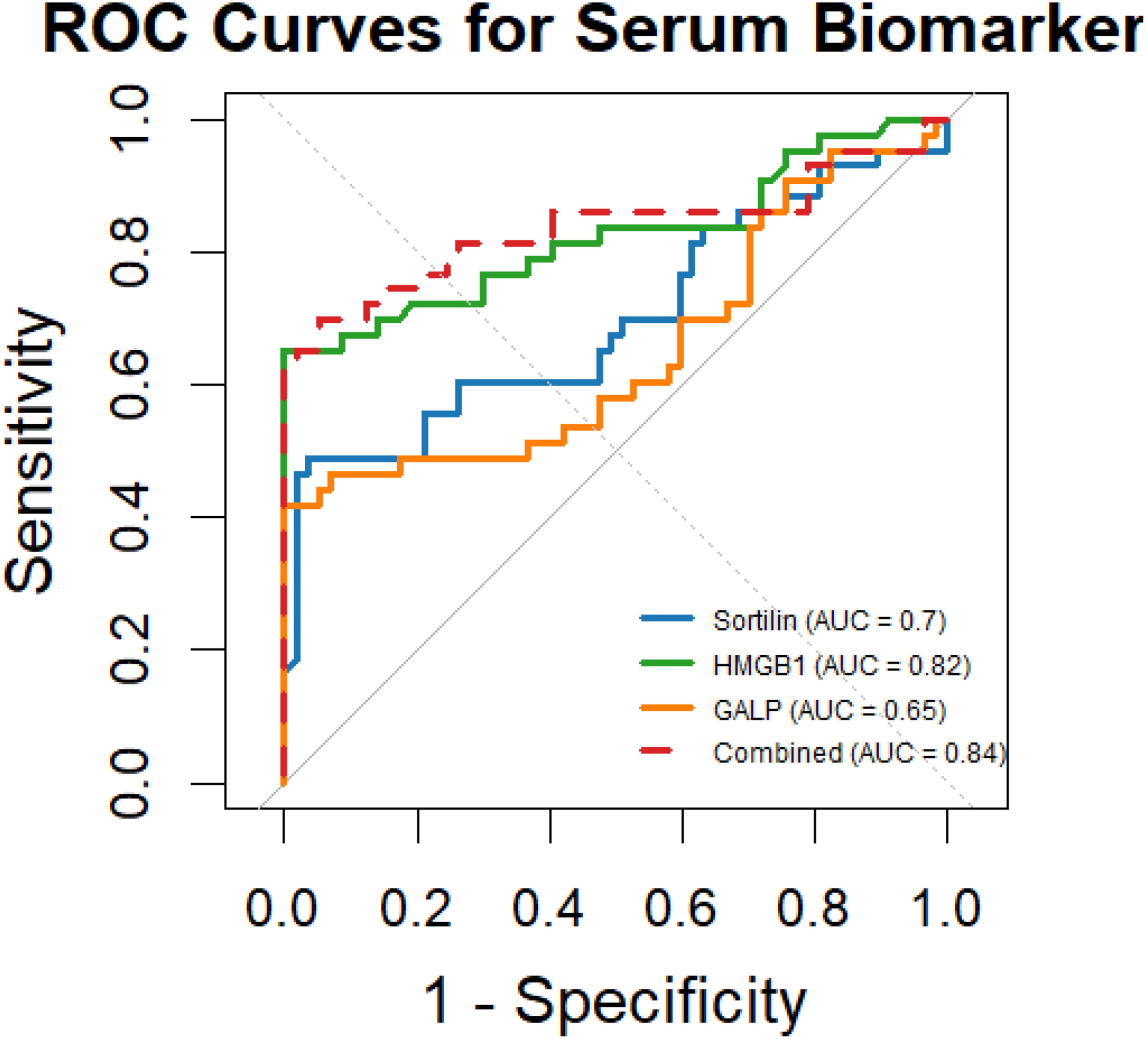
ROC curves showing the predictive performance of serum Sortilin, HMGB1, and GALP for GDM risk in PCOS pregnancies. AUC values are displayed in the figure for visual comparison of each biomarker’s discriminative ability. Corresponding p-values and Youden indices are detailed in **Table 3**. GALP, Galanin-Like Peptide; HMGB1, High Mobility Group Box 1.

ROC curve analysis was conducted to evaluate the discriminative ability of each serum biomarker for GDM prediction (**Figure 2**). The AUC represents the overall ability of the test to discriminate between patients who developed GDM and those who did not. The area under the curve (AUC) for Sortilin was 0.70 (95% CI: 0.57–0.82, p = 0.004), with an optimal cut-off value of 205.6 ng/mL, yielding a sensitivity of 72% and specificity of 63%. HMGB1 exhibited superior diagnostic performance, with an AUC of 0.82 (95% CI: 0.72–0.92, p < 0.001) and a cut-off value of 65.5 ng/mL, achieving 85% sensitivity and 75% specificity. GALP showed a modest predictive capacity, with an AUC of 0.65 (95% CI: 0.52–0.78, p = 0.012) and an optimal threshold of 73.3 pg/mL, corresponding to a sensitivity of 66% and specificity of 55%. Importantly, the combined multi-marker model significantly outperformed individual markers, achieving an AUC of 0.84 (95% CI: 0.74–0.94, p < 0.001), with 88% sensitivity and 78% specificity at the optimal probability threshold determined by logistic regression. These results underscore the clinical utility of GALP and HMGB1 as early predictive biomarkers, particularly when used in combination for risk stratification in PCOS pregnancies For clarity and clinical interpretability, AUC values were retained within the ROC plots to facilitate intuitive visual comparison between biomarkers, while detailed statistical metrics are provided in **Table 3**.

**Table 3:** Predictive value of serum markers for GDM risk among PCOS.

Indicator	AUC	95% CI		Sensitivity	Specificity	Youden Index	P value	Cut off Value
		CI Lower	CI Upper					
Sortilin	0.70	0.57	0.82	0.72	0.63	0.35	0.004	205.6 ng/mL
HMGB1	0.82	0.72	0.92	0.85	0.75	0.60	<0.001	65.5 ng/mL
GALP	0.65	0.52	0.78	0.66	0.55	0.21	0.012	73.3 pg/mL
Combined	0.84	0.74	0.94	0.88	0.78	0.66	<0.001	- (model)

GALP, Galanin-Like Peptide; HMGB1, High Mobility Group Box 1.

## Discussion

This study demonstrates that elevated serum GALP (aOR 1.55) and HMGB1 (aOR 1.65) independently predict GDM development in PCOS pregnancies, with combined biomarker assessment showing superior predictive accuracy (AUC 0.84). These novel markers outperformed conventional metabolic parameters, highlighting their potential for early risk stratification.

PCOS is a complex endocrine-metabolic disorder featuring ovulatory dysfunction, hyperandrogenemia, and insulin resistance (IR), with clinical manifestations including menstrual irregularities, infertility, and metabolic disturbances (38–40). Recognized as a major risk factor for GDM by leading guidelines, PCOS confers risk through synergistic mechanisms: pre-pregnancy IR compounded by pregnancy-induced hormonal changes (estrogen, progesterone, placental lactogen) that further impair insulin sensitivity (41–43). The magnitude of this risk has been quantified in multiple studies. A comprehensive meta-analysis demonstrated that PCOS patients face a 2.8-3.7 fold increased risk of developing GDM compared to healthy pregnant women (95% CI: 2.4-4.1) (44). The 2012 ASRM consensus statement further estimated that 40-50% of PCOS pregnancies are complicated by GDM (45). These findings underscore the critical need for enhanced screening and early intervention strategies in PCOS pregnancies to mitigate the substantial risk of GDM and its associated adverse outcomes. Future research should focus on elucidating precise molecular mechanisms and developing targeted preventive approaches for this high-risk population.

Sortilin is a multifunctional peptide hormone that plays a regulatory role in glucose and lipoprotein metabolism, insulin sensitivity, arterial wall inflammation, and calcification (30, 46). It is implicated in the pathogenesis of cardiovascular and metabolic diseases (47). Studies have demonstrated significantly elevated serum Sortilin levels in patients with diabetes mellitus, suggesting that elevated Sortilin levels may be associated with the onset of diabetes. In this study, serum Sortilin levels were markedly higher in patients with PCOS complicated by GDM compared to the control group. However, after adjusting for covariates, elevated Sortilin levels were not identified as an independent risk factor for GDM. Previous research has indicated that Sortilin plays a crucial role in regulating glucose homeostasis and insulin signaling (48). Sortilin and glucose transporter 4 (GLUT4) are co-expressed in adipocytes and muscle cells, where they are essential for glucose storage, transport, and homeostasis maintenance (49). Studies have shown that Sortilin knockout reduces diet-induced obesity and glycolysis in mice (50). Additionally, upregulated Sortilin expression has been observed in insulin resistance models, suggesting that Sortilin overexpression may be associated with glucose metabolism abnormalities and insulin resistance (51, 52). These findings imply that increased Sortilin expression may contribute to the pathogenesis of GDM by exacerbating glucose and lipid metabolism disorders and insulin resistance. However, the precise mechanisms underlying this relationship require further investigation.

Galanin-like peptide (GALP), a neuropeptide involved in energy metabolism and reproductive regulation, may influence the risk of GDM in PCOS patients through multiple mechanisms (34, 53). Research indicates that GALP modulates gonadotropin secretion via the hypothalamic-pituitary-ovarian (HPO) axis, while the characteristic LH/FSH ratio imbalance and hyperandrogenemia in PCOS patients may further exacerbate IR, a core pathogenic mechanism of GDM (34). Additionally, GALP enhances insulin sensitivity in adipose tissue and skeletal muscle, and its downregulation may contribute to glucose metabolism disorders, thereby increasing GDM susceptibility (53). Animal studies have demonstrated that GALP deficiency exacerbates high-fat diet-induced glucose intolerance, and the observed reduction in GALP levels in obese PCOS patients may promote chronic inflammation and lipid metabolism dysregulation, collectively driving GDM development (54). Although current evidence suggests a potential association between GALP and PCOS-related GDM, further clinical studies are required to elucidate its precise mechanistic role and evaluate its feasibility as either a predictive biomarker or therapeutic target.

HMGB-1, a potent proinflammatory protein, plays a pivotal role in inflammatory responses by promoting immune cell maturation, migration, and proinflammatory cytokine secretion (55). In pregnant women with PCOS, elevated HMGB-1 levels both reflect and exacerbate the chronic low-grade inflammatory state characteristic of this condition. Mechanistically, HMGB-1 contributes to GDM development by impairing insulin signaling cascades while simultaneously inducing pathological apoptosis in placental tissues. Furthermore, it amplifies inflammatory mediators through positive feedback mechanisms that directly compromise pancreatic β-cell function (56), thereby promoting insulin resistance (as quantified by HOMA-IR) and establishing a self-perpetuating cycle that progressively disrupts glucose homeostasis during pregnancy (57).

The integration of Sortilin, HMGB1, and GALP into a first-trimester predictive panel (AUC 0.84) enables a paradigm shift from reactive GDM diagnosis to proactive prevention in PCOS pregnancies. This multi-analyte approach identifies high-risk patients 12-16 weeks before conventional diagnosis, allowing timely initiation of pathophysiology-targeted interventions: metabolic management for Sortilin elevation, anti-inflammatory strategies for HMGB1, and endocrine modulation for GALP abnormalities. The panel’s clinical implementation could substantially reduce the 46% GDM incidence in this high-risk population by facilitating risk-stratified interventions ranging from intensified lifestyle counseling to pharmacologic prophylaxis.

### Limitations and future directions

Our study has several important limitations that warrant consideration. First, the single-center nature of this research, while ensuring procedural consistency, may limit the generalizability of our findings to broader populations with diverse ethnic, socioeconomic, or clinical backgrounds. Second, although our sample size (n = 139) met the requirements for primary statistical analyses, the moderate number of participants may have limited the power to detect subtle associations or perform more granular subgroup analyses. Third, the observational design of this study introduces the potential for selection bias, and residual confounding cannot be excluded. Although we adjusted for key covariates such as maternal age, BMI, and HOMA-IR, we lacked data on important lifestyle-related confounders, including detailed dietary intake, physical activity levels, and sleep quality, all of which may influence both biomarker levels and the risk of GDM. Fourth, biomarkers were measured only once in early pregnancy, which precludes the evaluation of dynamic longitudinal changes across gestation that could offer additional insights into the pathophysiological trajectory of GDM in PCOS. Finally, postpartum follow-up data were not collected, which limits our ability to assess whether these biomarkers are predictive of long-term metabolic outcomes beyond pregnancy.

Future studies should focus on validating these biomarkers in larger, multi-ethnic cohorts while incorporating serial measurements across pregnancy to better understand their temporal relationships with GDM development. Mechanistic studies are needed to elucidate how GALP and HMGB1 contribute to GDM pathogenesis in PCOS, potentially informing targeted prevention strategies. Translationally, research should prioritize developing clinically feasible assays and determining optimal cutoff values for risk stratification in diverse clinical settings.

## Conclusions

In conclusion, this study identified serum GALP and HMGB1 as independent early-pregnancy biomarkers associated with an increased risk of developing GDM in women with PCOS. Notably, the combined use of GALP and HMGB1 demonstrated superior predictive performance over traditional clinical indicators such as BMI and fasting glucose, suggesting their potential utility for early risk stratification. These findings underscore the promise of incorporating GALP and HMGB1 into clinical screening algorithms to enable earlier identification and intervention in high-risk PCOS pregnancies. However, given the single-center design and moderate sample size, further large-scale, multicenter prospective studies are needed to validate these biomarkers and establish standardized cutoff values for clinical application.

## Data Availability

The raw data supporting the conclusions of this article will be made available by the authors, without undue reservation.
